# Effect of Different Concentrations of Forskolin Along with Mature Granulosa Cell Co-Culturing on Mouse Embryonic Stem Cell Differentiation into Germ-Like Cells

**DOI:** 10.29252/ibj.24.1.30

**Published:** 2019-08-28

**Authors:** Soghra Bahmanpour, Amirhesam Keshavarz, Nehleh Zarei Fard

**Affiliations:** Laboratory for Stem Cell Research, Department of Anatomical Sciences, School of Medicine, Shiraz University of Medical Sciences, Shiraz, Iran

**Keywords:** Embryonic stem cells, Forskolin, Germ cells, Granulosa cells

## Abstract

**Background:**

Germ cell development processes are influenced by soluble factors and intercellular signaling events between them and the neighboring somatic cells. More insight into the molecular biology of the germ cell development from ES cells and investigation of appropriate factors, specifically those targeting differentiation processes, is of great importance. In this study, we established an *in* *vitro* model with higher ES cell differentiation rate to germ cells, using adenylate cyclase activator, forskolin.

**Methods:**

ES cells were first cultured for five days, leading to EB formation. Subsequently, the EB were dissociated and cultured for an additional three days in different forskolin concentrations of 5, 20, and 50 µM, with or without GC co-culture. On the 8^th^ day, we analyzed the expressions of 5 germ cell-specific markers using quantitative real-time-PCR technique along with cell viability assay by MTT test.

**Results:**

Our results showed that in the GC-free cultures, a 50-µM concentration of forskolin resulted in a significant increase in *Mvh*, *Gdf9*, *Scp3*, and *Rec8* expression levels in comparison to the control. However, when the cells were co-cultured with the GCs, 20-µM concentration of forskolin could also increase the expression of those germ cell-specific marker genes. Furthermore, results from the MTT assay showed enhanced cell proliferation and survival at all three concentrations of forskolin, but 20-µM concentration was the most potent one.

**Conclusion:**

These data indicate that forskolin can stimulate differentiation and proliferation, dose-dependently; however, the influence of GCs *co-culturing *should not go unnoticed.

## INTRODUCTION

An interesting area of germ cell biology is to *understand* the molecular mechanisms of germ cell specification and development. In this sense, an extensive research should be carried out to identify suitable factors that specifically trigger germ cell differentiation process. One strategy to study gametogenesis is by inducing germ cell differentiation from the ES cells *in* *vitro*. 

Several studies have reported the expression of germ cell-specific genes in ES cells using various factors, such as RA, bone morphogenetic protein 4, and basic fibroblast growth factor^[^^1^^-^^3^^]^. The positive effects of ES cell co-cultivation with GCs or other somatic cells have also been investigated^[^^2^^,^^4^^-^^7^^]^. Recently, it has been reported that the combinatorial application of 10 µm forskolin and rolipram, which both increase the cAMP levels, can stimulate the proliferation and development of the primordial germ cell-like cells derived from the mouse ES cells^[^^8^^]^. 

It has been postulated that *adenylate cyclase activation *by the non-physiological activator *forskolin* is accountable for synthesis of the second messenger cAMP. The adenylate cyclase-cAMP system can induce various biological and biochemical effects on different cells, depending on the cell type and the applied concentration^[^^9^^]^. In this regard, the role of cAMP in initiating meiosis in germ cells, regulation of oocyte maturation, and cell proliferation has been documented^[^^10^^-^^15^^]^. On the other hand, researches have shown that increased cAMP levels in the denuded oocytes and cumulus cell-enclosed oocytes in response to forskolin exposure can induce or block meiosis via different pathways^[^^16^^-^^18^^]^. Although the role of forskolin has been recognized in the development of germ cells from ES cells^[^^8^^]^, there has been no investigation on the suitable dose of forskolin to induce germ cell differentiation from ES cells. The processes of *in* *vitro* ES cells differentiation to germ cell-like cells might be different from primordial germ cells development within *fetal gonads*. 

As a priority, it is important to identify the suitable dose of exogenous factors inducing germ cell differentiation from ES cells with higher frequency. In this study, the effects of three different concentrations of forskolin on germ cell differentiation were investigated. In addition, we designed two approaches of induction of germ cell differentiation including with or without co-culturing the cells with GCs, and then the germ cell markers were examined to determine the rate of germ cell differentiation in each method.

## MATERIALS AND METHODS

**ES culture**

Undifferentiated mouse ES cell line R1^[^^19^^]^ was cultured on inactivated mouse embryonic fibroblasts in KnockOut D-MEM medium (Gibco, UK) supplemented with 15% ES cell-qualified FBS, 1% penicillin/streptomycin, 2 mM of L-glutamine, 0.1 mM of non-essential amino acids (all from Gibco), 0.1 mM of β-mercaptoethanol (Sigma, UK), and 1000 U/mL of leukemia inhibitory factor (Chemicon, USA) and incubated in a humidified atmosphere containing 5% CO_2_ at 37 °C. Half of the medium was changed daily, and the cells were passaged every two days. 

**GC culture**

Ovarian GCs were obtained from the ovaries of adult BALB/c mice based on the method provided by Sèdes^[^^20^^]^, with minor modifications. Briefly, following the collection of ovaries from adult mice, trimming was done to omit extra tissues 48 h after gonadotropin administration. First, the ovaries were exposed to 6.8 mM of EGTA and 0.2% BSA in the DMEM/F12 medium (Shelmax, Iran) at 37 °C for 20 min. Then the ovaries were placed in a hypertonic solution (0.5 M of sucrose, 1.8 mM of EGTA and 0.2% BSA) in DMEM/F12 at 4 °C for 5 min. After that, the ovaries were punctured with an insulin needle, and the cells were pelleted by a 5-min centrifugation at 1200 rpm. The collected cells were seeded in DMEM/F12 with 10% FBS and 1% penicillin/streptomycin and maintained in a 5% CO_2_ incubator at 37 °C overnight. The remaining oocytes were then separated from the attached GCs through GC subculture. GCs were used as a feeder layer at the second passage in 24-well culture plates pre-coated with 0.1% gelatin at a density of 5 × 10^4^ cells/well (Jet Biofil, Korea), after inhibiting the proliferation by incubating with 10 µg/mL of mitomycin C.

**ES cell differentiation**

**Fig. 1:**
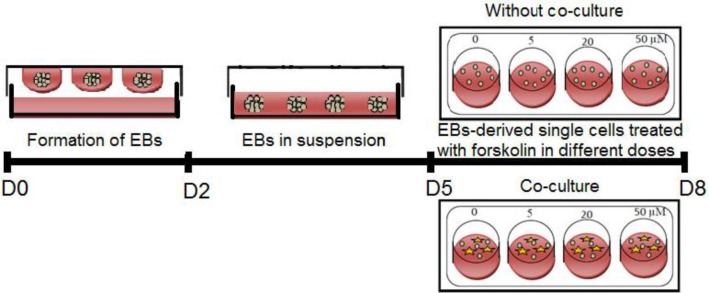
Schematic presentation of the experimental protocol used for the ES cell-derived germ-like cells by treatment of cells at different concentrations of forskolin in the presence or absence of granulose feeder layer

ES cells were isolated from the feeder layer by the incubation of single cell suspensions on gelatin-coated tissue culture plates at 37 °C for 1-1:30 h and differentiated as EB through hanging drop method. For hanging drop culture, the isolated ES cells were seeded in leukemia inhibitory factor-free ES medium at a density of 2000 cells/20 µL for 2 days. Thereafter, the *cells* were collected and transferred into non-adhesive plates and cultured until the 5^th^ day. After EB formation, EB-derived differentiation cells were dissociated and cultured at a density of 5 × 10^4^ or 10^5^ cells/well (24-well culture plate), with or without GC-derived feeder cells, respectively. The germ cell differentiation media were DMEM/F12 supplemented with 10% FBS, 2 mM of L-glutamine, 0.1 mM of β-mercaptoethanol, and 0.1 mM of non-essential amino acids with 5 µM, 20 µM, or 50 µM concentrations of forskolin (Sigma) as the experimental groups or without treatment as the control group (Fig. 1). The cells were cultured in these media for three more days, and half of the differentiation media were changed on day two. Morphological modifications were monitored daily by an invert microscope (Olympus, UK) equipped with a Nikon DXM-1200C digital camera. All analyses were performed on day eight of ES cell differentiation protocols. To be sure about the probability of germ cell expression markers by GCs, these cells were seeded in the same media alone for gene expression assessment. 

**Quantitative real-time -PCR analysis**

Total RNAs were isolated from the experimental groups, using the Biazole reagent (Bioflux, Japan). Genomic DNA contamination was eliminated by DNase I, and cDNA was prepared in a total volume of 20 µL via the cDNA synthesis kit (Fermentase, Lithuania), based on the manufacturer's instructions. Expression and quantity of *Stra8*, *Scp3*,* Rec8*, *Mvh*, and *Gdf9* genes were assessed using the SYBR Green I PCR Master Mix (Applied Biosystems, USA) containing 150 nmol of each forward and reverse primer (Table 1). The annealing temperature was 57 °C, and the running cycles were 40 on an Applied Biosystems 7500 System. Target gene expression was normalized by the housekeeping gene β-actin.

**MTT assay**

Viability of germ-like cells differentiated from ES cells in the presence of various *forskolin concentrations* *in the no co-culture* *group* was analyzed via *MTT (Sigma, UK) assay*. Accordingly, cells were incubated with 500 µL of 5 mg/mL MTT for 4 h, followed by an elution of the dye with 200 µL of DMSO (Sigma, USA). Thereafter, the cells were transferred into a 96-well plate, and their absorbance rate was measured at a 595 nm wavelength, using a microplate reader (BioPhotometer plus Eppendorf, Germany).

**Statistical analysis**

Results were presented as mean ± SD. Statistical analyses were performed using One-way ANOVA, and the post-hoc test of LSD in SPSS 22.0 for windows (SPSS, Chicago, USA), and the graphs were plotted via the Prism v6.07 software (GraphPad software, USA). A *p* value <0.05 was regarded to be statistically significant.

## RESULTS

We examined the expression of germ cell markers by exposing ES cells to different concentrations of forskolin in the presence/absence of GCs, aiming to determine whether forskolin is involved in the regulation of germ cell differentiation from ES cells. We assessed the possibility for expression of germ cell markers by GCs, used for co-culture groups, by the evaluation of mRNA levels, and the result showed negligible expression levels of these markers.

**Fig. 2:**
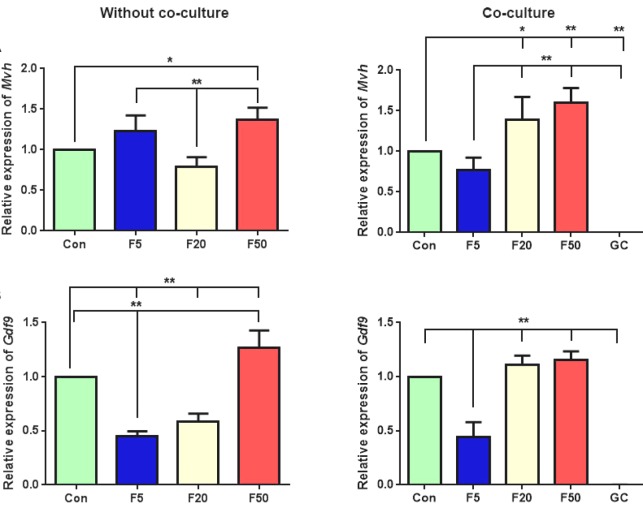
QRT-PCR analysis of (A) *Mvh* and (B)* Gdf9* expression in differentiated cells after treatment with different forskolin (F) concentrations (5 µM, 20 µM, and 50 µM), with or without GC co-culture. The gene expression was also analyzed for GC cultures alone. The results were presented relative to control RNA level. Data are presented as means ± SD (n =3). ^*^p < 0.05, ^**^p < 0.01; Con, control

We found that in the absence of GCs, *Mvh* expression, as a germ cell-specific gene, significantly enhanced by adding 50 µM of forskolin in comparison with the control condition. The concentration of 5 µM of forskolin increased *Mvh *expression in EB*-*derived differentiated cells, but it was not significant compared with the control (*p* = 0.069). Also, a significant increase in *Mvh* expression was observed in both high concentrations of forskolin (20 and 50 µM), using the co-culture method (*p* = 0.03 and *p* = 0.004, Fig. 2A). 

**Table 1:** The sequences of specific primers used in this study

**Gene**	**Forward primer (5'-3')**	**Reverse primer (5'-3')**
*Stra8*	GCATGAAGGACAGCGGCGTG	AAAGGATCTCTTCTGGGGTGGACTC
*Scp3*	GCAGAGAGCTTGGTCGGGGC	CTTTAGATGTTTGCTCAGCGGCTCC
*Rec8*	GCCCTAGAAGGTGCTGGTTTGG	GTGGGGTCACCTCAGTGAGTAGG
*Mvh*	CAAGCGAGGTGGCTGCCAAG	CTGAATCACTTGCTGCTGGTTTCC
*Gdf9*	CGTCCGGCTCTTCAGTCCCT	CCATCGGCAGCGGTCCTGTC
*-actin* *β*	CCCGCGAGCACAGCTTCTTTG	CCATCACACCCTGGTGCCTAGG

The expression level of *Gdf9*, as a post-meiotic marker, significantly elevated at 50 µM concentration of forskolin in the absence of granulosa feeder layer (*p* = 0.006). Furthermore, there was a significant reduction in *Gdf9* expression at 5 and 20 µM forskolin concentrations* compared with those in the control* cultures (*p* = 0.0001, *p* = 0.0004, respectively). It should be noted that when *EB*-*derived differentiated cells* were co-cultured with the GCs, *Gdf9* expression was slightly, *but not significantly* higher at 20 and 50 µM forskolin concentrations than the control group (*p* = 0.7, *p* = 0.2, respectively, Fig. 2B).

**Fig. 3:**
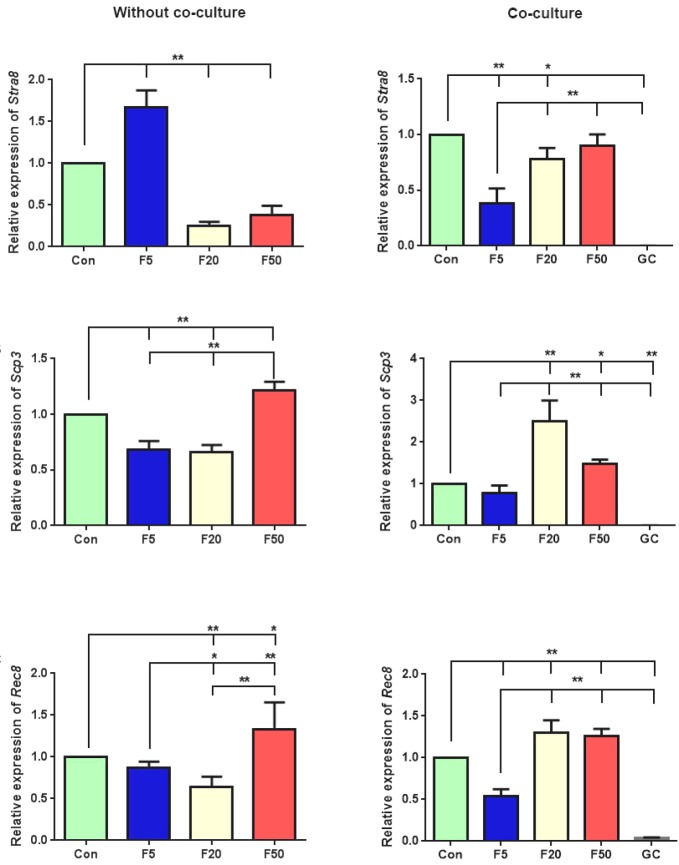
Gene expression analysis of meiotic markers, including (A) *Stra8*, (B) *Scp3*, (C) *Rec8* in differentiated cells after treatment with three different forskolin (F) concentrations (5, 20, and 50 µM), with or without GC co-culture. GCs were cultured alone for controlling these gene expression patterns. Values are means ± SD (n = 3). ^*^p < 0.05, ^**^p < 0.01; Con, control

In the absence of GCs, the expression of *pre-meiotic gene* *Stra8 *increased significantly compared with the control group in concentration of 5 µM (*p* = 0.0001). In contrast, in the co-culturing groups, its expression significantly reduced at 5 and 20 µM concentrations (*p* = 0.00005, *p* = 0.02, respectively) and was not affected by 50 µM of forskolin (*p* = 0.2, Fig. 3A). Moreover, without co-culturing the cells with GCs, the expression of meiotic genes *Scp3* and *Rec8* significantly elevated at the 50 µM concentration of forskolin (*p* = 0.003 and *p* = 0.01, respectively). Interestingly, when the co-culture method was applied, *Scp3* expression increased significantly at both 20 and 50 µM concentrations *in comparison* to the control (*p* = 0.00001 and *p* = 0.036, respectively). Meanwhile, similar results were obtained in *Rec8* expression at 20 and 50 µM concentrations (*p* = 0.001 and *p* = 0.003, respectively, Fig. 3B and 3C). 

**Fig. 4:**
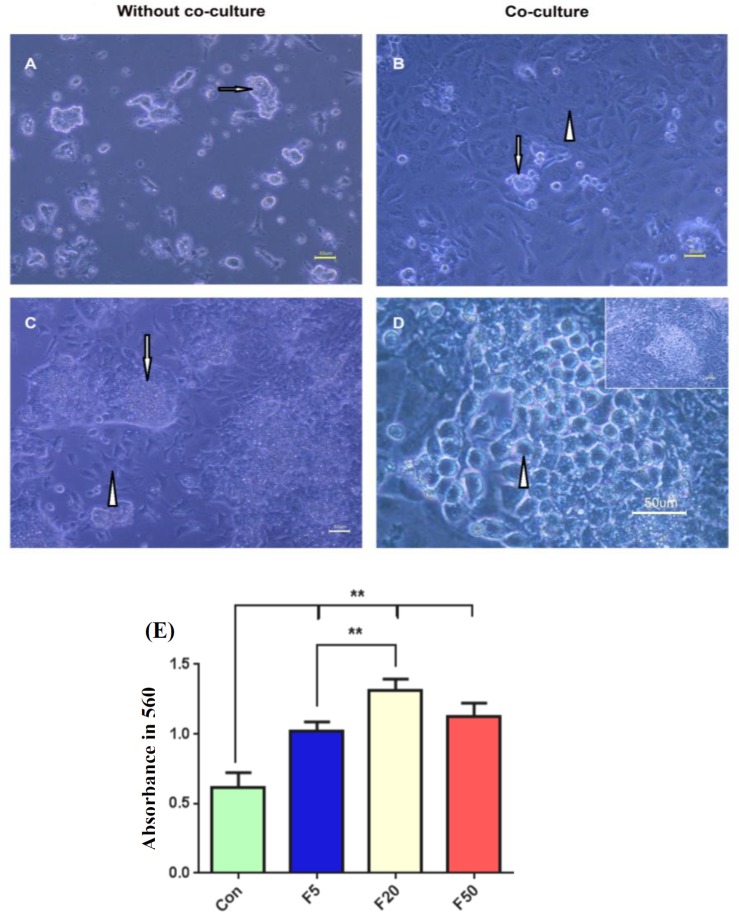
Morphological assessment of differentiated cells after treatment in various concentrations of forskolin in the absence (A and C) or presence (B and D) of the GC co-culturing. The *phase-contrast microscopy* revealed that differentiated colonies (arrows) in day one were surrounded by (C) flat-shape cells (arrowhead) in the absence of the GCs in day three. The differentiated cells manifested as (D) round and large germ-like cells (arrowhead) around the colonies (arrow) when cultured on the granulosa feeder layer (B, arrowhead). Scale bars, 50 µm. (E) Cell viability assay in differentiated cells cultured in the presence of different concentrations of forskolin (F). Data are presented as means ± SD (n = 3). ^**^p < 0.01; Con, control

In addition, the morphological observations revealed that both culturing methods had led to cell proliferation and formed colonies with similar morphology to those in ES cells (Fig. 4A and 4B). However, after day two, the round cells appeared only around the colonies in all experimental groups at approximately *the same cellular density* in the co-culture method, which were considered as germ-like cells in the size range of 10-15 µM. In contrast, no round cells were observed in cultures without the granulosa feeder layer, and the colonies were less attached to the culture plates in this method that were surrounded by flat-expanded cells (Fig. 4C and 4D).

Cell viability was shown via MTT assay. The results reveled that forskolin significantly induced higher cell proliferation in all concentrations *in comparison* to the same *cells cultured* in the absence of forskolin (*p* < 0.001), while 20 µM concentration was more effective (*p* = 0.00001, Fig. 4E).

## DISCUSSION

Exploring the molecular mechanism of germ cell development is crucial for both basic and clinical research. In the present study, we showed that forskolin can *dose-dependently* regulate germ cell differentiation from ES cells. Aforementioned data indicates the presence of *forskolin-stimulated *Mvh* and *Gdf9* expressions, which was dose dependent. *Mvh* expression is considered as the germ cell-specific marker*, and Gdf9* plays a critical role in folliculogenesis*^[^^21^^,^^22^^]^. Inducing cAMP signaling has been found. To induce early gametogenesis and oocyte development^[^^12^^,^^14^^,^^15^^]^. According to *our findings, *high concentration of forskolin (50 µM) can *be considered alone as* another factor influencing germ cell differentiation from ES cells. In addition, the current study showed increased gene expression of the meiotic genes *Rec8 *and *Scp3. In a previous study, it has been confirmed* *that* exposure *to a* 20-µM forskolin concentration can induce meiotic prophase progression in the organ culture of the fetal rabbit ovaries^[^^23^^]^. Moreover, increased cAMP concentration from 15.5 to 17.5 days post coitum was actively involved in establishing the primordial follicle pool and inducing early meiosis in the mice ovaries^[12]^. Another study has reported that 20 µM of forskolin concentration triggered meiosis in the germ cells derived from 11.5 days post coitum XY genital ridges cultured in *vitro*, as it led to the increased expression of synaptonemal complex proteins^[^^15^^]^. In contrast, increased cAMP levels caused a delay in meiosis II progression in mature and immature oocytes, while it was able to regulate the oocyte developmental competence^[^^14^^,^^18^^,^^24^^]^. Hence, in the present study, *Rec8 *and *Scp3 *gene expressions in EB-derived differentiated cells through forskolin treatment indicated meiosis entry.

In the absence or presence of GC feeder layer, *Stra8* expression elevated only at concentration of 5 µM of forskolin, and other concentrations had no effect on the expression of this gene. There is strong and extensive evidence indicating the increased *expression* of Stra8 induced by RA in the germ cells after entering the genital ridges^[^^25^^,^^26^^]^. The induction of meiosis was reported by the presence of RA in the serum (FBS) introduced to the medium^[^^27^^]^. It seems that the low concentration of forskolin in the possible presence of approximately 3.6 × 10^-8^M RA^[^^28^^]^ in the 10% FBS supplemented medium was able to stimulate Stra8 *expression* as a pre-meiotic gene, whereas higher forskolin doses inhibited the expression of this RA-responsive gene. Interestingly, a previous study has indicated that BMP4 and RA treatment of cultured spermatogonia increases *Stra8* expression in comparison with RA stimulation alone^[^^29^^]^. On the other hand, *Stra8* expression is inhibited by the expression of *Cyp26b1*, *a member of the* cytochrome p450 superfamily^[^^27^^]^, and in GCs, Cyp26b1 mRNA is expressed at all developmental stages^[^^30^^]^. The results of our study showed that forskolin had no effect on inducing *Stra8* expression in the co-culturing method.

The presence of round cells around colonies, *known as* a germ cell-like population, was revealed only after co-cultivation with GCs. In line with our finding, other studies have suggested the role of newborn ovarian GCs in oocyte-like cell differentiation from ES cells^[^^1^^,^^4^^]^*.* In the mouse embryo, Saiti *et al.*^[^^31^^]^ could distinguish primordial germ cells from the adjacent somatic cells by their large oval or round shape in the size range of 10 µM after gonad colonization, which was *consistent with our* observations. Besides, in our study, germ cell-specific markers, such as *Mvh*, *Gdf9*,* Scp3*, and* Rec8 *demonstrated increased expression at 20 µM forskolin concentration after co-culturing with GCs, but not without co-culturing. Several studies have reported that GCs secrete various soluble factors into the culture medium and play a significant role in gametogenesis^[^^32^^-^^35^^]^. GCs are the main sources of cAMP, purines*/pyrimidines*, metabolites, and amino acids for oocytes, and these substances are transferred through the gap junctions. These small regulatory molecules are essential for growth and maturation of germ cells and oocytes^[^^36^^]^. In this regard, paracrine interactions between germ cells and the neighboring somatic cells by kit ligand/stem cell factor have been found to critically function in both female and male germ cell development^[^^37^^]^. Thus, these observations *can be* attributed to *cross talks* between GCs *and* *EB*-*derived dissociated cells.* Moreover, the results of other studies have indicated forskolin ability to enhance the production of estrogen, progesterone, and cAMP in the cultured GCs in a dose-dependent manner that serves as a non-hormonal stimulator of these cells^[^^38^^,^^39^^]^. 

To determine the effect of forskolin on cell viability, we cultured cells differentiated from ES cells in various concentrations of forskolin for 72 h. Under these conditions, forskolin treatment was able to increase cell proliferation in a dose-dependent manner. The optimal concentration of forskolin to enhance proliferation was at 20 µM. Some studies have depicted that different concentrations of forskolin (especially *20* μM) could enhance cell proliferation. It is believed that forskolin-induced cAMP synthesis*,* as a potent mitogen, is a major factor for optimal germ cell growth^[^^10^^,^^40^^-^^42^^]^, which is in contrast to others that declared treatment with forskolin resulted a decrease in cellular viability^[^^43^^,^^44^^]^. 

In summary, our findings revealed that forskolin-mediated stem cell differentiation is dependent on dose and the type of cell culture (with or *without *GC *co-culture)*. According to the expression of germ cell specific markers, forskolin can induce germ-like cell differentiation directly and through co-cultured GCs indirectly. However, in co-culture method, which has been used as the most successful system for induction of germ cell differentiation in previous *studies*, 20 µM of forskolin concentration is suitable, and higher concentrations are not *required*.
